# Glucagon‐Like Peptide‐1 Targets in the Human Nodose Ganglion

**DOI:** 10.1002/cne.70135

**Published:** 2026-01-31

**Authors:** Warda Merchant, Claire Mackaaij, Cindy G. J. Cleypool, Laurent Gautron

**Affiliations:** ^1^ Department of Internal Medicine and Center for Hypothalamic Research UT Southwestern Medical Center Dallas Texas USA; ^2^ Division of Surgical Specialties, Department of Anatomy University Medical Center Utrecht The Netherlands

**Keywords:** autonomic nervous system, endocrinology, Glp1r, *Homo sapiens*, human, in situ hybridization, nodose ganglion, peripheral afferent neurons

## Abstract

Given the rapidly expanding clinical use of glucagon‐like peptide‐1 receptor (GLP1R) agonists—well‐known for their antidiabetic and antiobesity effects—it is increasingly important to understand the precise distribution of GLP1R expression in the human body, as this knowledge is crucial for elucidating both their therapeutic effects and side effects. In this study, we investigated *Glp1r* mRNA expression in the human nodose ganglion, a key sensory relay between the periphery and the brain. We analyzed postmortem paraffin‐embedded nodose ganglia sections from 10 human donors, using RNAscope analysis. We found that optimal tissue required fixation times under 48 h and postmortem intervals of approximately 10 h or less. Ultimately, nine nodose ganglia from six donors met quality standards for analysis. Using multiplex RNAscope, we detected moderate to high levels of *Glp1r* expression in approximately 7% of all nodose neurons, with no clear differences between sides, sex, or age. The proportion of neurons with low *Glp1r* expression rose to nearly 28%. Notably, *Glp1r* expression was also observed in nonneuronal cells within the perineurium, epineurium, and fascicles of the human vagus nerve. As a point of comparison, we also examined *Glp1r* expression in mice, where 17.9%–29.1% of nodose neurons were positive, with slightly higher expression on the right side. In mice, *Glp1r* expression was strictly neuronal. Overall, our findings demonstrate that the human nodose ganglion is a potential target for GLP1R‐based therapeutics and reveal species similarities and differences in *Glp1r* expression between humans and mice.

## Graphical abstract

Our findings demonstrate that the human nodose ganglion expresses *Glp1r* mRNA and reveal species similarities and differences in *Glp1r* expression between humans and mice.

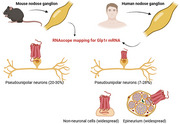

## 1 Introduction

The glucagon‐like peptide‐1 receptor (GLP‐1R) is the target of widely used antiobesity and antidiabetes medications (Cook et al. 2025; Drucker and Holst 2023), with millions of individuals currently receiving GLP‐1R‐based therapies. Identifying the cell types and anatomical sites that mediate the clinical effects of GLP‐1 receptor agonists is crucial for elucidating their mechanisms of action and potential side effects (Brierley et al. 2021; Burmeister et al. 2017; Dumiaty et al. 2024; Kim et al. 2024; Sisley et al. 2014). Much of our current anatomical knowledge regarding GLP‐1R stems from studies in laboratory rodents, where its distribution has been extensively characterized in the brain, pancreas, and gastrointestinal tract (Alvarez et al. 1996; Cantacorps et al. 2023; Cork et al. 2015; de Bray et al. 2025; Greiner et al. 2024; Lund et al. 2018; Richards et al. 2014; Smith et al. 2022; Yamamoto et al. 2003; Zheng et al. 2019).

Interestingly, *Glp1r* transcript enrichment has also been observed in the nodose ganglion of rats (Nakagawa et al. 2004). Supporting this, electrophysiological studies have shown increased firing of rodent vagal afferent neurons following administration of GLP‐1 or its peptide analogs (Bucinskaite et al. 2009; Gaisano et al. 2010). More recently, high‐throughput qPCR, single‐cell RNA sequencing, and RNA in situ hybridization have confirmed that *Glp1r* mRNA is enriched in subsets of vagal sensory neurons within the mouse nodose ganglion (Bai et al. 2019; Egerod et al. 2018; Lansbury et al. 2025; Lowenstein et al. 2023; Welch et al. 2025; Williams et al. 2016). *Glp1r*‐expressing vagal sensory neurons innervate the muscularis of the gastrointestinal tract and are responsive to mechanical deformation and suppress food intake (Bai et al. 2019; Borgmann et al. 2021; Williams et al. 2016). Interestingly, *Glp1r* mRNA expression is widespread throughout the nodose ganglion, suggesting that there may be unique subtypes of *Glp1r*‐expressing neurons that are performing unique roles based on their expression of other receptors or their projection patterns.

Despite these findings, the physiological role of vagal GLP‐1R signaling itself within vagal sensory neurons remains debated. Some studies support a central role for vagal pathways in mediating GLP‐1's clinical effects (Krieger et al. 2015; Ruttimann et al. 2009; Varin et al. 2019), while others challenge this view (Bethea et al. 2025; Huang et al. 2024; Williams et al. 2016). Nonetheless, the presence of GLP‐1R in the nodose ganglion is notable, as these vagal ganglion cells are not protected by a blood–brain barrier and regulate many physiological functions associated with the clinical effects of GLP‐1, including feeding and glucose homeostasis (Berthoud et al. 2021). Thus, *Glp1r*‐expressing vagal sensory neurons may represent a functionally relevant site of action for peripherally administered GLP‐1R agonists. Alternatively, these neurons might contribute to unwanted side effects, such as nausea, altered gastrointestinal motility, and visceral discomfort (Borner et al. 2021; Holmes et al. 2009).

The extent to which these rodent findings translate to humans remains unknown. In particular, the study of visceral primary sensory neurons in the nodose ganglion has lagged behind that of somatosensory primary sensory neurons, especially regarding studies of human tissue. While *Glp1r* mRNA and GLP1R protein have been successfully detected in the human brain and various peripheral tissues (Alvarez et al. 2005; Baggio et al. 2018; Korner et al. 2007; Ten Kulve et al. 2016; Thorens et al. 1993; Tornehave et al. 2008), their expression in the human nodose ganglion has, to our knowledge, not been investigated. Although certain sensory pathways are evolutionarily conserved, notable species‐specific differences in sensory neuron biology have been reported. For example, comparative transcriptomic analyses of human and mouse dorsal root ganglia (DRG) revealed substantial differences in gene expression profiles and neuronal subtype organization (Nguyen et al. 2021; Ray et al. 2018; Rostock et al. 2018; Shiers et al. 2020). Similarly, in the hypothalamus, *Glp1r* does not exhibit the same clustering patterns in humans as observed in mice (Tadross et al. 2025), further underscoring potential species divergence. These observations raise the possibility that vagal sensory neuron properties, including *Glp1r* expression patterns, may also differ between rodents and humans. However, the study of human vagal sensory neurons is limited by the rarity of human nodose ganglion tissue available for research.

In this study, we sought to address this critical knowledge gap by analyzing postmortem human nodose ganglia obtained via a human body donation program. First, we evaluated tissue quality using histological methods to determine the suitability of formalin‐fixed, paraffin‐embedded (FFPE) samples, obtained from cadavers with varying postmortem intervals (PMIs), for mRNA in situ hybridization (RNAscope). We then characterized the distribution of *Glp1r* mRNA within the human nodose ganglion. Finally, we compared these findings with data from the mouse nodose ganglion to identify conserved features and species‐specific differences.

## 2 Materials and methods

### 2.1 Human Sample Approval, Collection, and Preparation

Nodose ganglia were resected from 10 adult human cadavers. Bodies were donated through a body donation program to the Department of Anatomy at the University Medical Center Utrecht (UMCU), the Netherlands. Informed consent was obtained during life, allowing the use of these bodies for educational and research purposes. The study was reviewed and approved by the departmental research committee, and no additional medical–ethical approval was required. Table 1 contains details of each cadaver used (age, gender, and PMIs). To access the nodose ganglia, the head was separated from the body just above the clavicular bones. The head was then placed upside down, allowing exposure of the carotid sheaths in the transverse plane. The vagus nerve was identified on each side, gently grasped with forceps, and carefully dissected free from the surrounding tissues using fine, long, blunt scissors. The dissection proceeded cranially until the skull base could be palpated with the scissors. The vagus nerve was then slightly tensioned and transected at the skull base using a long, fine scalpel. If the vagus nerve and nodose ganglion were not yet fully released from adjacent structures, additional blunt dissection and gentle separation were performed. Following removal, the nodose ganglion was verified by its increased thickness and firmer consistency compared to the vagus nerve and was separated from the nerve. Subsequently, all ganglia were placed in tissue cassettes and fixed in 4% formaldehyde (21–48 h) and processed for standard paraffin embedding by placing them in increasing percentages of ethanol and xylene, followed by immersion in paraffin. After paraffin embedding, each ganglion was cut serially in 8‐µm‐thick sections using a microtome (Leica Histocore Autocut R, Nussloch, Germany). Ten serial sections of 10 levels (with 48 µm between levels) of each ganglion were then mounted onto glass slides (Superfrost Plus, VWR, Leuven). All sections were air‐dried and heat‐fixed for 2 h on a slide drying table at 60°C (Medax, 14801, Kiel, Germany), followed by overnight heat fixation in an incubation oven at 60°C (Binder, Tuttlingen, Germany).

**TABLE 1:** Summary of human donor demographics and sample characteristics. Formalin‐fixed and paraffin‐embedded sections were 8‐µm thick. A total of 10 sections per NG (48 µm interval) were collected and used for either FastRed or Multiplex RNAscope. Donor #82 had a permanent glucose sensor, indicating a diabetic status.

Sample ID#	Sex	Age (years)	PMI (h)	Fixation (h)	Side
12	**♀**	76	48	42	L
13	♂	80	48	24	L, R
14	**♀**	90	11	40	R
16	♂	86	72	27	L
17	**♀**	85	10	24	L
18	**♀**	85	9.5	21	R
19	♂	71	Unknown	23	L, R
32	♂	70	8	24	L, R
66	**♀**	79	22	24	L, R
82	♂	80	9	24	L, R

Abbreviations: L, left; PMI, postmortem interval; R, right.

### 2.2 Mouse Sample Collection and Preparation

C57BL/6J male and female mice were obtained from the Jackson lab (stock# 000664) (RRID:IMSR_JAX:000664). Animals were group‐housed in a barrier facility with ad libitum access to food and water in a temperature‐controlled room (∼23°C) with a 12:12‐h light–dark cycle. Mice were approximately 6–10 weeks old at the time of euthanasia. The UT Southwestern Medical Center Institutional Animal Care and Use Committee approved our methods for terminal euthanasia. Briefly, mice received an overdose of chloral hydrate (500 mg/kg, i.p.) before being intracardially perfused with 1x phosphate‐buffered saline (PBS), followed by 10% formalin (room temperature). Vagal ganglia were collected with a dissecting scope and fine spring scissors, postfixed overnight at 4°C, and then transferred to a solution of 30% sucrose for 24 h. Samples were frozen on dry ice and cut with a cryostat into sections of 14–16 µm thickness.

### 2.3 RNAscope

The specificity of antibodies used to detect GLP‐1R protein has been questioned, raising concerns about the reliability of immunohistochemical data (Drucker 2013; Wong et al. 2025). Thus, we opted for the detection of *Glp1r* mRNA using RNAscope, a method with a proven record of high specificity and sensitivity in a variety of tissue samples, including mouse, rat, and human tissues (Bono et al. 2022; Hall et al. 2022; Hultman et al. 2019; Sapio et al. 2020). While most samples were processed with the standard protocol recommended by Advanced Cell Diagnostics (ACD) (RRID:SCR_012481), a few modifications were necessary when working with human samples, as explained below.

#### 2.3.1 Chromogenic RNAscope Applied to Human Samples

PPFE slides were deparaffinized by baking them for 1 h at 60°C, followed by two 5‐min incubations in xylene and two 1‐min washes in 100% ethanol. Slides were then air‐dried. Subsequently, slides were processed according to the ACD protocol using reagents from the RNAscope 2.5 HD Chromogenic Detection Kit (FastRed). Pretreatment conditions were optimized for each sample, taking into account factors such as PMI and fixation duration. The pretreatment involved boiling the slides in retrieval buffer at approximately 99°C for 30 min. Protease Plus digestion was performed for either 30 min (standard) or 45 min (extended), depending on the sample characteristics (see Section 3). The Snap25 probe was hybridized to the tissue sections for 2 h at 40°C using the ACD hybridization oven (see Table 2). Slides were counterstained with hematoxylin. FastRed signals, indicative of *Snap25* expression, appeared as large red dots under brightfield microscopy. Since *Snap25* signals initially accumulated in nearly all neurons, and nonneuronal cells and fiber tracts lacked any red signal, we concluded that our assay was highly specific. Of note, lipofuscin accumulation was commonly seen as a brownish crescent in the cytoplasm of many neuronal profiles. However, lipofuscin did not interfere with our ability to visualize FastRed signals under brightfield illumination.

**TABLE 2:** In situ hybridization (ISH) Z probes for human and mouse with information available from the ACD website.

Probe (dyes)	Cat. No.	Accession ID	Target region (bp)	Use
Hs‐Snap25 (opal 520 or FastRed)	518851	NM_003081.4	797–2066	Pan‐neuronal marker
Mm‐Snap25 (opal 520)	516471	NM_011428.3	215–1498	Pan‐neuronal marker
Mm‐Phox2b (opal 690)	407861‐C2	NM_008888.3	1617–2790	Marker of nodose neurons
Hs‐Glp1r (opal 570)	519821‐C3	NM_002062.3	1583–2841	Receptor for GLP‐1
Mm‐Glp1r (opal 570)	418851‐C3	NM_021332.2	108–1203	Receptor for GLP‐1

#### 2.3.2 Multiplex Fluorescent RNAscope Applied to Human and Mouse Samples

PPFE slides (human) were deparaffinized and pretreated as described above. To minimize lipofuscin autofluorescence commonly observed in aging sensory neurons (Sapio et al. 2025; Shiers et al. 2020), slides were exposed for 30 min to high‐power illumination using the TiYo quenching system (Nepa Gene Co. Ltd, Chiba, Japan). In this system, slides are immersed in PBS and illuminated with high‐power, broad‐spectrum white light LED arrays (>3000 lumens/LED). It was previously shown to be effective at reducing lipofuscin artifacts within 15–30 min of illumination (Tsuneoka et al. 2022). Immediately after illumination, samples were boiled in retrieval buffer at approximately 99°C for 30 min. Thereafter, samples were digested with Protease Plus for either 30 min (standard) or 45 min (extended), depending on each sample's characteristics (see Section 3). Reagents were all included in the ACD RNAscope multiplex kit (cat. 323110). Probes listed in Table 1 were hybridized to the tissue for 2 h at 40°C using the ACD hybridization oven. Probes were labeled by using opal dyes (Akoya Biosciences, Japan).

Mouse tissues were formalin‐fixed and did not require deparaffinization or autofluorescence quenching. Pretreatment and hybridization procedures followed the standard RNAscope protocol with a 15‐min boiling step and a 30‐min pretreatment step. Probes are listed in Table 2. Since fluorescent signals were distributed exactly as expected based on prior anatomical studies of the mouse nodose ganglion, we concluded that our assay was highly specific. All samples (human and mouse) were counterstained with DAPI to visualize nuclei and mounted using ProLong Gold Antifade Mounting Medium (Invitrogen, cat#P36934). Additional methodological details are available in our previous publication (Bob‐Manuel and Gautron 2021).

### 2.4 Data Analysis and Production of Digital Photomicrographs

Photomicrographs of FastRed‐stained ganglia were acquired using a DM6B‐Z (Leica) brightfield microscope equipped with LAS X software (RRID:SCR_013673). Fluorescent imaging was performed on a Zeiss LSM980 confocal microscope (RRID:SCR_025048) equipped with 405, 488, 561, and 639 laser lines for imaging DAPI, Opal 520, Opal 570, and Opal 690, respectively. Laser power and gain settings were optimized to minimize nonspecific background fluorescence by first imaging unlabeled sections until no or low background fluorescence was detected and lipofuscin autofluorescence was minimal. These settings were then applied consistently across all samples. Preferred objectives included 10x (for tile scans), 20x, and 63x oil immersion lenses. Images were acquired using Zen software with line averaging set to 4 and a minimum pixel resolution of 1024 × 1024. Digital images were exported at 300 dpi and processed using ImageJ Fiji (NIH, win64 version) (RRID:SCR_002285). Final figure assembly, including cropping, resizing, auto‐contrast adjustment, and labeling, was performed uniformly in Adobe Photoshop 2024.

Chromogenic signals for *Snap25* were analyzed using brightfield digital images in ImageJ Fiji. Hematoxylin‐stained vagal sensory neuron cell bodies were readily identified and outlined using the selection tool. Surface area (µm^2^) and signal intensity (dots) were measured for each selected cellular profile. Approximately three to five fields of view at 20x magnification were analyzed per sample. Data were plotted in GraphPad Prism 10 to illustrate the relationship between preparation parameters (PMI and fixation) and *Snap25* signal intensity. Likewise, the gap between *Snap25*‐positive perikarya and the endoneural parenchyma was quantified using the measurement tools in ImageJ Fiji. Neuronal density was assessed by counting positive neurons and dividing by the area of each field of view using ImageJ Fiji. Correlations between PMI and measured parameters were analyzed by fitting a standard linear regression line in GraphPad Prism.

Using digital images of fluorescently labeled samples, lipofuscin fluorescence was quantified with ImageJ Fiji. Areas containing lipofuscin were outlined using the selection tool (three cells per sample, in three samples). Mean gray values were measured and compiled for each channel. In addition, the percentage of positive profiles expressing each transcript of interest was calculated.

Cells were considered *Glp1r* positive when at least one brightly labeled dot was observed within one *Snap25*‐positive profile (human) or *Phox2b*‐positive profile (mouse) somata. *Glp1r*‐positive neurons were spatially distinct and easily discernible. Digital images (single plane) were opened in ImageJ Fiji. For each identified *Snap25*‐positive profile, the numbers of *Glp1r*‐positive dots were manually counted. In parallel, the intensity of Snap25 signals was estimated by outlining each profile and using the measuring tool (mean gray value). To account for inherent variability in sample quality, data were normalized by calculating the ratio of the number of *Glp1r*‐positive dots to the mean gray values. Cells were arbitrarily categorized as low, moderate, and highly expressing when ratios were above 0, 0.15, and 0.3, respectively. Counting was repeated in four sections per sample. Numerical data are presented as mean ± standard error of the mean (SEM) and were compiled using GraphPad Prism 10. Cell counts from human samples were analyzed using a *t*‐test function (two‐tailed and equal variance) performed in Excel. Cell counts from mouse samples were analyzed using a two‐way ANOVA (with sex and side as factors), followed by a post hoc uncorrected Fisher's LSD test in GraphPad Prism.

## 3 Results

### 3.1 Troubleshooting of Human Samples

**FIGURE 1:**
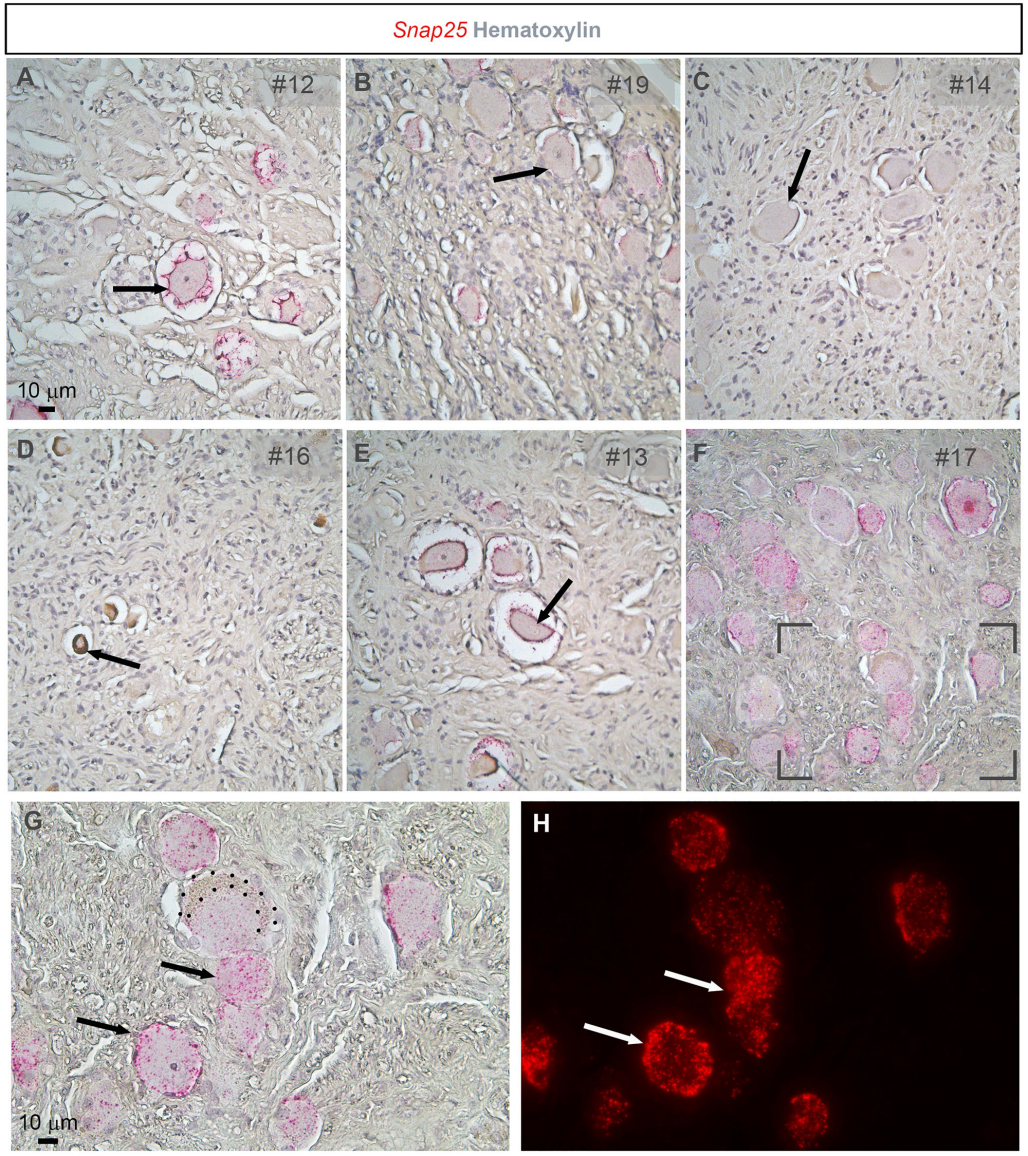
Troubleshooting chromogenic in situ hybridization for *Snap25* (FastRed dots) in the human nodose ganglion. *Snap25*, a pan‐neuronal marker, exhibited variable expression depending on the characteristics of the human samples analyzed. (A–E) Examples of samples showing neurons with poor morphology (star shaped, shriveled, tear‐drop shaped) and low or absent *Snap25* signals. These samples generally corresponded to tissue with a prolonged postmortem interval (PMI) and/or extended fixation duration. (F–H) Example of a sample displaying acceptable neuronal morphology with clearly visible *Snap25* signals. The inset in panel (F) corresponds to panel (G). Image (H) was captured using epifluorescence microscopy. Arrows indicate representative neurons. Sample numbers are indicated in the top right corner of each image. Tissue sections were counterstained with hematoxylin. Scale bar in (A) applies through (F), and the scale bar in (G) applies to (H).

Samples from human donors were used for chromogenic RNAscope targeting *Snap25*, a pan‐neuronal marker, to assess which samples were best suited for RNAscope assays (Figure 1). We encountered various artifacts in the samples depending on their PMI and fixation times. Samples with 48 h of fixation and/or long PMIs displayed poor morphology, with vagal afferent neurons appearing shriveled and exhibiting a large pericellular gap (Figure 1A,B). In these samples, *Snap25* signals accumulated predominantly at the membrane rather than within the cytoplasm. Samples with a long fixation of 48 h (e.g., #14) exhibited good neuronal morphology but no discernible *Snap25* signals under standard pretreatment conditions (Figure 1C). Additionally, some samples with long PMI contained neurons that appeared dead (very small and brownish) (Figure 1D) or dying (tear‐drop‐shaped) (Figure 1E). The above features are reminiscent of dying neurons seen in the injured human brain and characterized by “vacuolation of the cytoplasm, shrinkage, or loss of their affinity for hematoxylin (ghost neurons)” (Mena et al. 2004). Based on the above analysis, samples with a PMI over 40 h (#12, #13, #16) were not further used. In addition, sample #19 had an unknown PMI and, given its low *Snap25* expression, was not included in our analysis. In contrast, samples fixed for 24 h and with a short PMI (∼10 h) showed well‐preserved neuronal morphology (Figure 1F), with abundant and uniform *Snap25* signals accumulating throughout the neurons (Figure 1G,H). No unwanted signals were detected outside the neuronal cell bodies.

**FIGURE 2:**
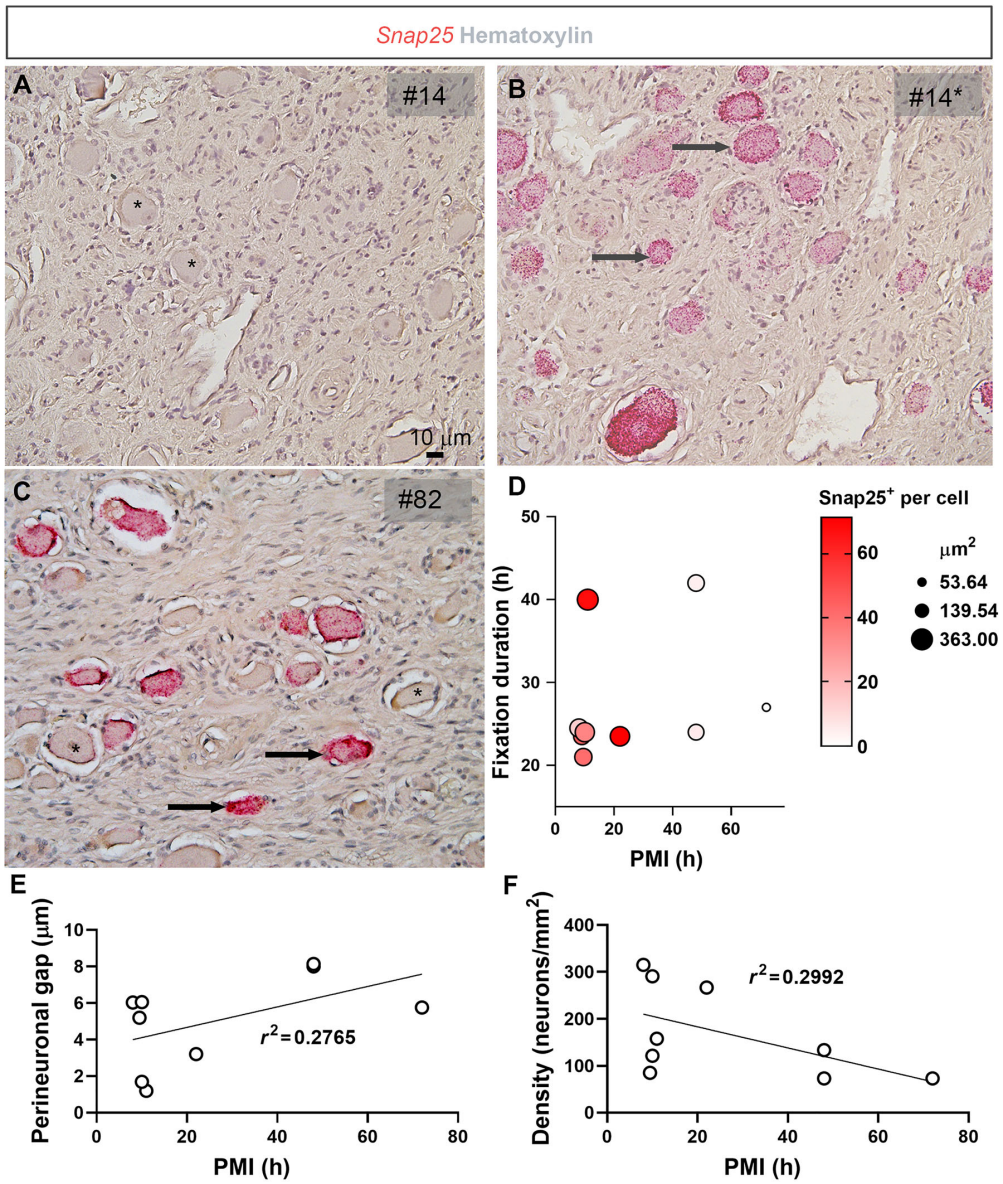
*Snap25* detection and neuronal morphology are influenced by postmortem interval (PMI) and fixation duration. (A) Detection of *Snap25* mRNA using chromogenic RNAscope (red dots) was suboptimal in sample #14 (long fixation time of 40 h). (B) Extended enzymatic digestion improved *Snap25* signal detection in sample #14. (C) Example of a sample displaying heterogeneous staining: some neurons showed strong *Snap25* labeling, while others were negative and exhibited poor morphology. (D) Graphical representation of factors affecting neuronal morphology and *Snap25* signal density. Samples with a PMI greater than 40 h consistently showed small, poorly preserved neurons and undetectable *Snap25* expression. In contrast, in samples with prolonged fixation (>30 h), *Snap25* signals could be partially recovered by extended pretreatment. Sample #16 was not plotted because we did not obtain its PMI. Arrows and asterisks indicate representative neurons with high and low (or absent) signals, respectively. Sample numbers are shown in the top right corner of each image. Tissue sections were counterstained with hematoxylin. Scale bar in (A) applies to panels (A–C). (E) Perineuronal gap (µm) and (F) density of neurons relative to the PMI of each sample. A fitted line was added, and the *r* ^2^ values were indicated.

Of note, *Snap25* signals went from undetectable to abundant in sample #14 after extended digestion (45 min) (Figure 2A,B). Thus, this sample was used with extended digestion. In addition, sample #32 was an outlier since it contained both well‐labeled neurons interspersed with shriveled and *Snap25*‐negative neurons (Figure 2C). Apart from the latter sample, overall correlations between PMI, fixation, cell size, and *Snap25* expression levels revealed that samples with a fixation between 24 and 48 h (provided adjustment in pretreatment) and short PMIs were optimal for high‐quality RNAscope assays (Figure 2D).

The gap between neuronal perikarya and the surrounding parenchyma was more pronounced in samples with longer PMIs (Figure 2E). However, below a PMI of 24 h, no strong correlation between PMI and gap size was observed. Similarly, neuronal density declined sharply in samples with the longest PMI (Figure 2F). Overall, neuronal density showed only a weak correlation with PMI. Based on the observations above, the following samples were deemed suitable for further analysis: #14, #17, #18, #66, and #82. Sample #32 was included with reservations.

### 3.2 General Anatomical Features of the Human Nodose Ganglion

**FIGURE 3:**
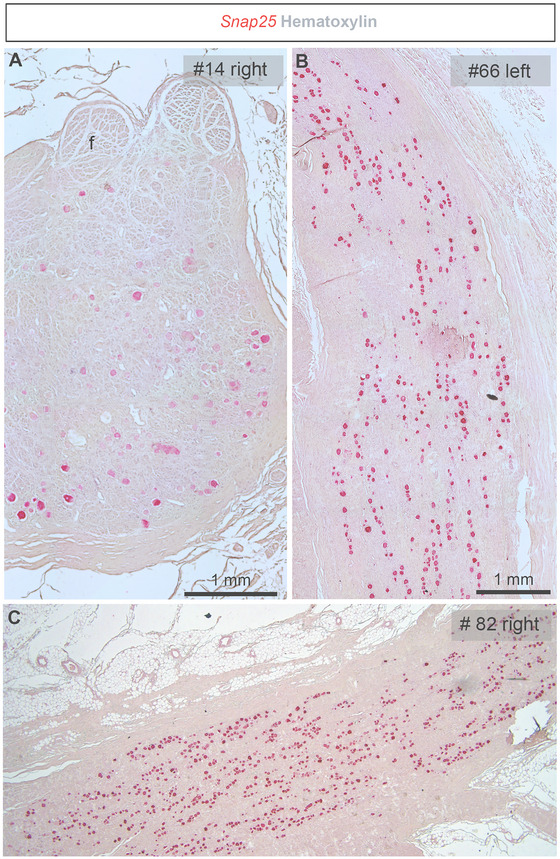
Morphology and cellular features of the human nodose ganglion. Detection of *Snap25* mRNA using chromogenic RNAscope (red dots) in representative human samples. (A) The transverse section shows a thick epineurium and fascicular organization of the vagal system. (B, C) Longitudinal sections reveal the elongated structure of the human nodose ganglion. Notably, neurons in human samples are spaced farther apart compared to those in rodent ganglia. The human nodose ganglion is often surrounded by thick layers of adipocytes. Sample numbers are indicated in the top right corner of each image. Tissue sections were counterstained with hematoxylin. Scale bar in (A) applies to panels (A–C).

At the morphological level, the human nodose ganglion displayed distinct features compared to that of rodents (Figure 3A–C). Notably, it exhibited a more fascicular organization, with a well‐developed epineurium, perineurium, and intraganglionic fibrous tissue (Figure 3A). Consequently, *Snap25*‐positive neuronal cell bodies in the human nodose ganglion were more widely spaced. In transverse sections, neurons were seemingly randomly distributed (Figure 3A), whereas in longitudinal sections, neurons often appeared as “strings of beads” (Figure 3B). The human nodose ganglion measured approximately 14.5 mm in length and 2.2 mm in width. Lastly, we noticed a significant accumulation of adipose tissue around the ganglion of most specimens (Figure 3C).

### 3.3 Multiplex Detection of *Glp1r* Expression in the Human Nodose Ganglion

**FIGURE 4:**
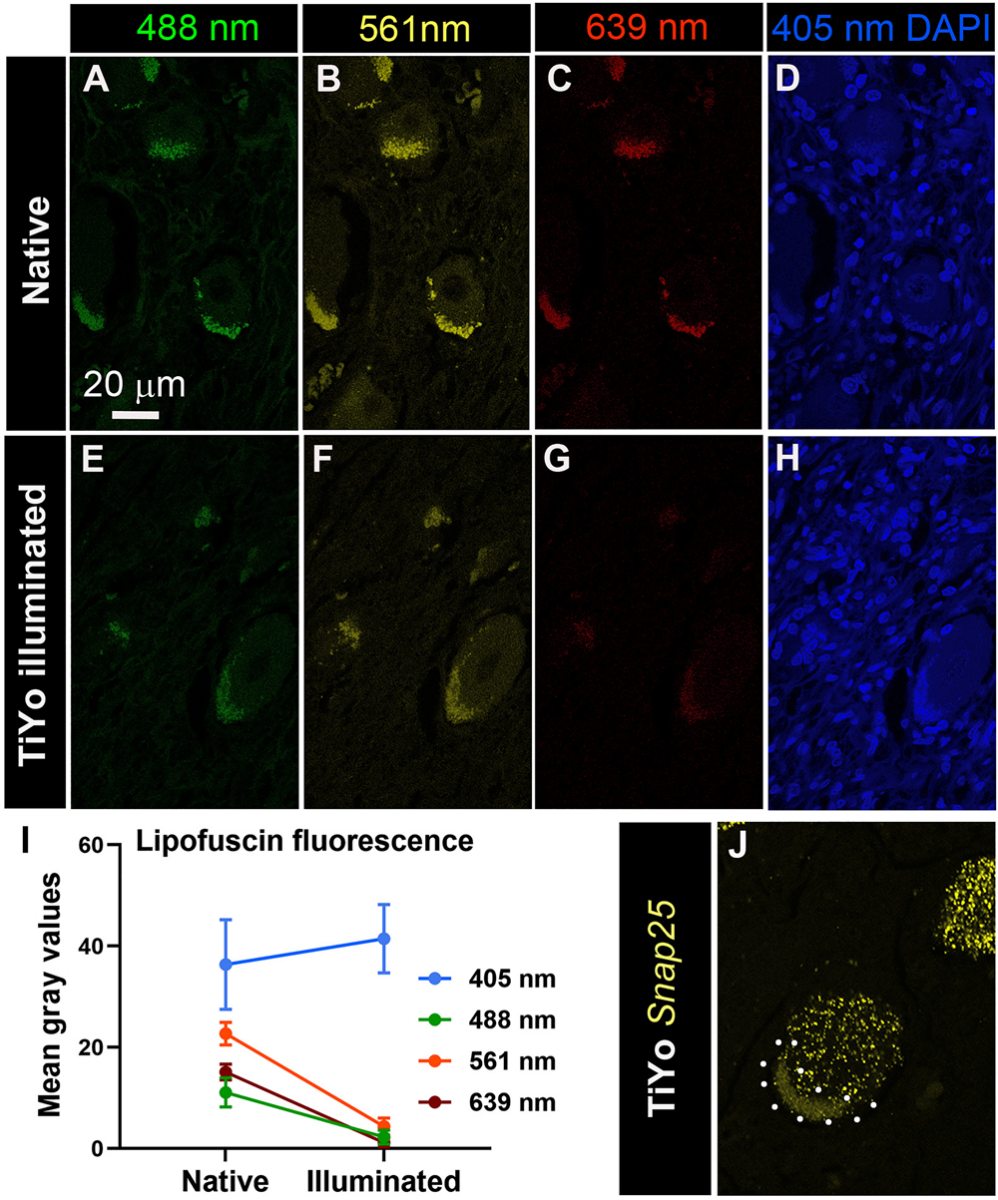
Lipofuscin autofluorescence artifact is reduced by high‐power illumination. Images were acquired using confocal microscopy (Zeiss LSM980) on samples that were not processed for RNAscope unless otherwise noted. (A–D) Native (unstained) tissue sections exhibited strong autofluorescence due to lipofuscin, a pigment that accumulates in neurons and appears as crescent‐shaped structures in the cytoplasm. Lipofuscin emitted across all excitation wavelengths. (E–H) Pretreatment with illumination using a TiYO system for 30 min prior to DAPI counterstaining reduced lipofuscin autofluorescence intensity, although some signal remained detectable. (I) Quantification of mean gray levels (± SEM, three samples per group) across channels showed that illumination effectively reduced autofluorescence in all wavelengths except the DAPI channel. (J) RNAscope detection of *Snap25* mRNA (yellow puncta) clearly labeled neuronal cytoplasm. Residual lipofuscin was still visible under RNAscope signals as pale, crescent‐shaped structures (outlined with a dotted line), but its intensity remained lower than that of the RNAscope signal. Scale bar in (A) applies to all panels.

Before assessing *Glp1r* expression, we assessed the suitability of our samples for multiplex fluorescent RNAscope. Unfortunately, all samples displayed lipofuscin artifacts in many neurons, with lipofuscin fluorescence being seen in all channels (Figure 4A–D). While lipofuscin could not be eliminated, high‐power illumination helped reduce fluorescence in all channels except the one used to visualize DAPI (Figure 4E–I). This is because DAPI was applied after illumination. Provided high‐power illumination and a choice of settings to minimize capturing background fluorescence, the lipofuscin artifact did not interfere with our ability to observe fluorescent RNAscope signals. Specifically, lipofuscin appeared as a crescent‐shaped halo of lesser intensity than RNAscope fluorescent dots (Figure 4J). The analysis presented below is based on this optimized protocol.

**FIGURE 5:**
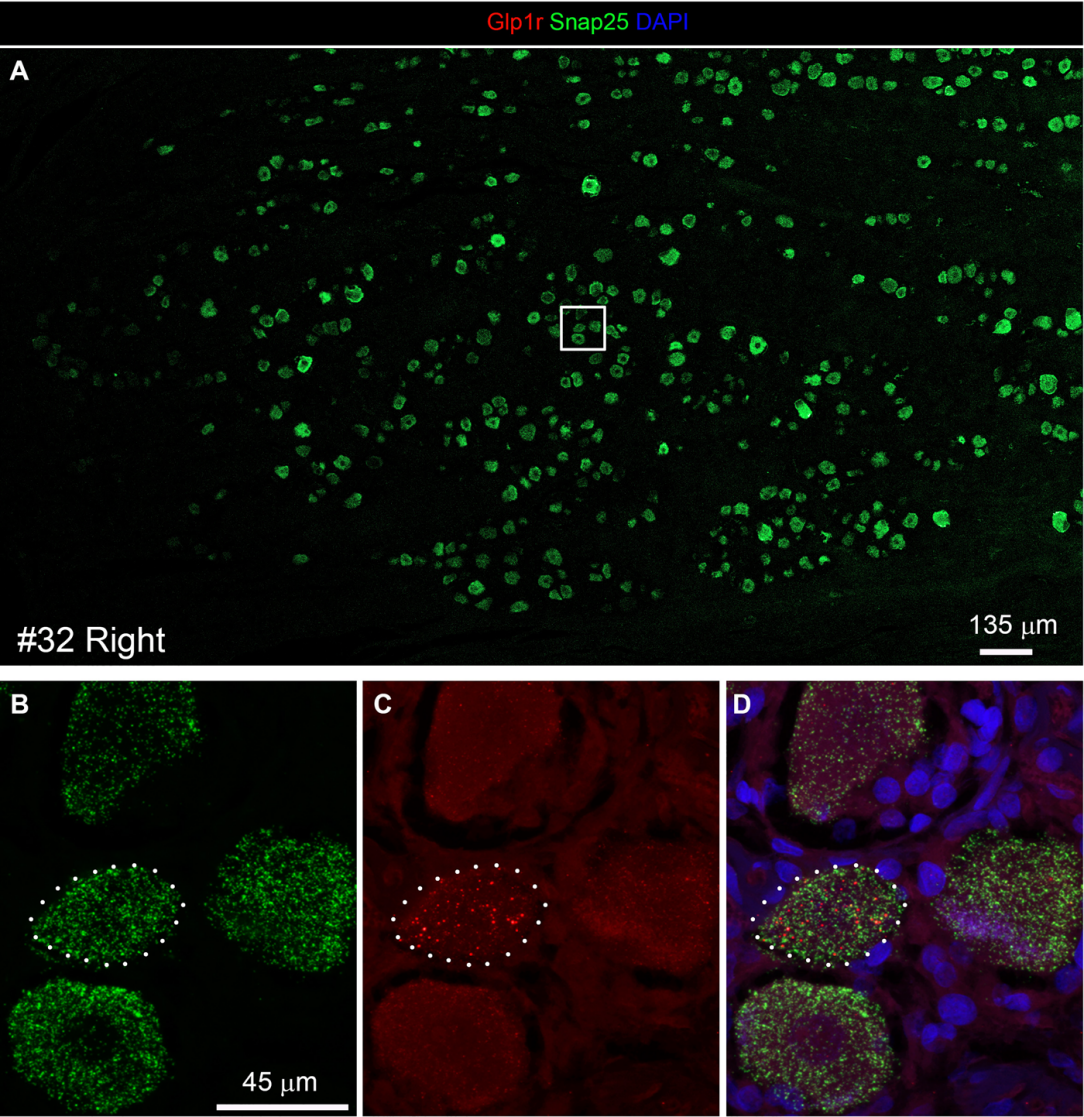
Representative confocal images showing *Glp1r* mRNA expression in the human nodose ganglion of sample #32 (right side). Samples were processed using multiplex RNAscope for *Snap25* (green), *Glp1r* (red), and DAPI (blue) and imaged with a Zeiss LSM980 confocal microscope. (A) Low‐magnification view (10x objective and tiles) of a cluster of vagal neurons. The inset is shown at higher magnification (63x oil objective) in panels (B–D). One *Snap25*‐positive neuron, outlined with a dotted line, exhibits high *Glp1r* expression. Other neurons display low (a few puncta) or no detectable signal. Scale bar in panel (B) applies to panels (B–D).

Within the ganglion itself, neurons exhibited varying levels of *Glp1r* expression. A subset of neurons showed strong signals, typically consisting of many RNAscope dots distributed across the cell body, aligning with the shape of the neuron (Figure 5A–D). *Glp1r*‐positive neurons were not necessarily near one another, nor were they confined to a specific region of the ganglion.

**FIGURE 6:**
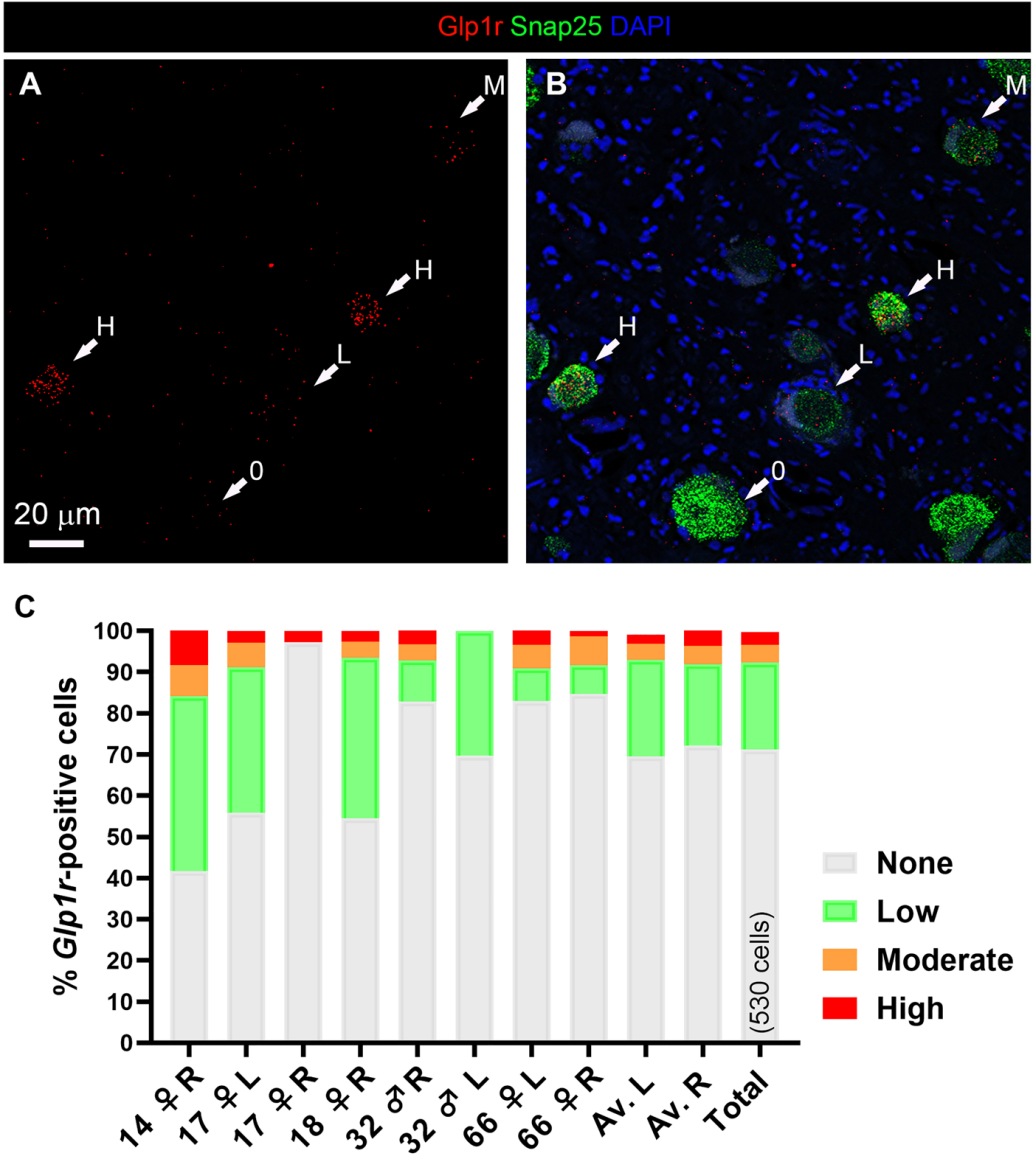
Estimates of the percentage of *Glp1r*‐expressing neurons in the human nodose ganglion. Representative confocal images showing neurons with varying levels of *Glp1r* expression in the human nodose ganglion. Samples were processed using multiplex RNAscope for *Snap25* (green), *Glp1r* (red), and DAPI (blue) and imaged with a Zeiss LSM980 confocal microscope. The dotted outlines represent *Snap25*‐positive vagal profiles. Cells were categorized as expressing no Glp1r or low, moderate, or high levels of Glp1r, based on the number of RNAscope dots per profile (normalized by Snap25 signal; see Section 2). (C) Percentage of neurons categorized by *Glp1r* expression level in each sample. 0, no expression; L, low expression; M, moderate expression; H, high expression. Scale bar in panel (A) applies to panel (B).

Neurons were classified into several categories based on their individual *Glp1r* expression levels (Figure 6A,B). Neurons exhibiting high and moderate expression levels showed clear positive signals, with numerous dots decorating their somata. In contrast, neurons with low *Glp1r* expression displayed fewer dots per cell profile (Figure 6A,B). However, most neurons exhibited low to no *Glp1r* signal (Figure 6A,B). Cell counting could not be reliably performed on sample #32 due to sparse and inconsistent expression of both *Snap25* and *Glp1r*. All other samples showed consistent *Glp1r* expression, except for sample #32 on the left side (Figure 6C). The percentage of *Glp1r*‐expressing neurons varied significantly between samples, ranging from a maximum of 16% of all neurons (#14 right) to as low as 3% (#17 right). There was no apparent correlation between *Glp1r* expression and factors such as sex or anatomical side. Due to the limited number of samples, we were unable to perform a comprehensive comparison between males and females. Overall, cell counting of over more than 500 neurons revealed high to moderate *Glp1r* expression in approximately 7% of neurons. However, when including neurons with low expression, the total proportion of *Glp1r*‐expressing neurons increased to approximately 39%.

### 3.4 Human *Glp1r* Expression in Nonneuronal Cells

**FIGURE 7:**
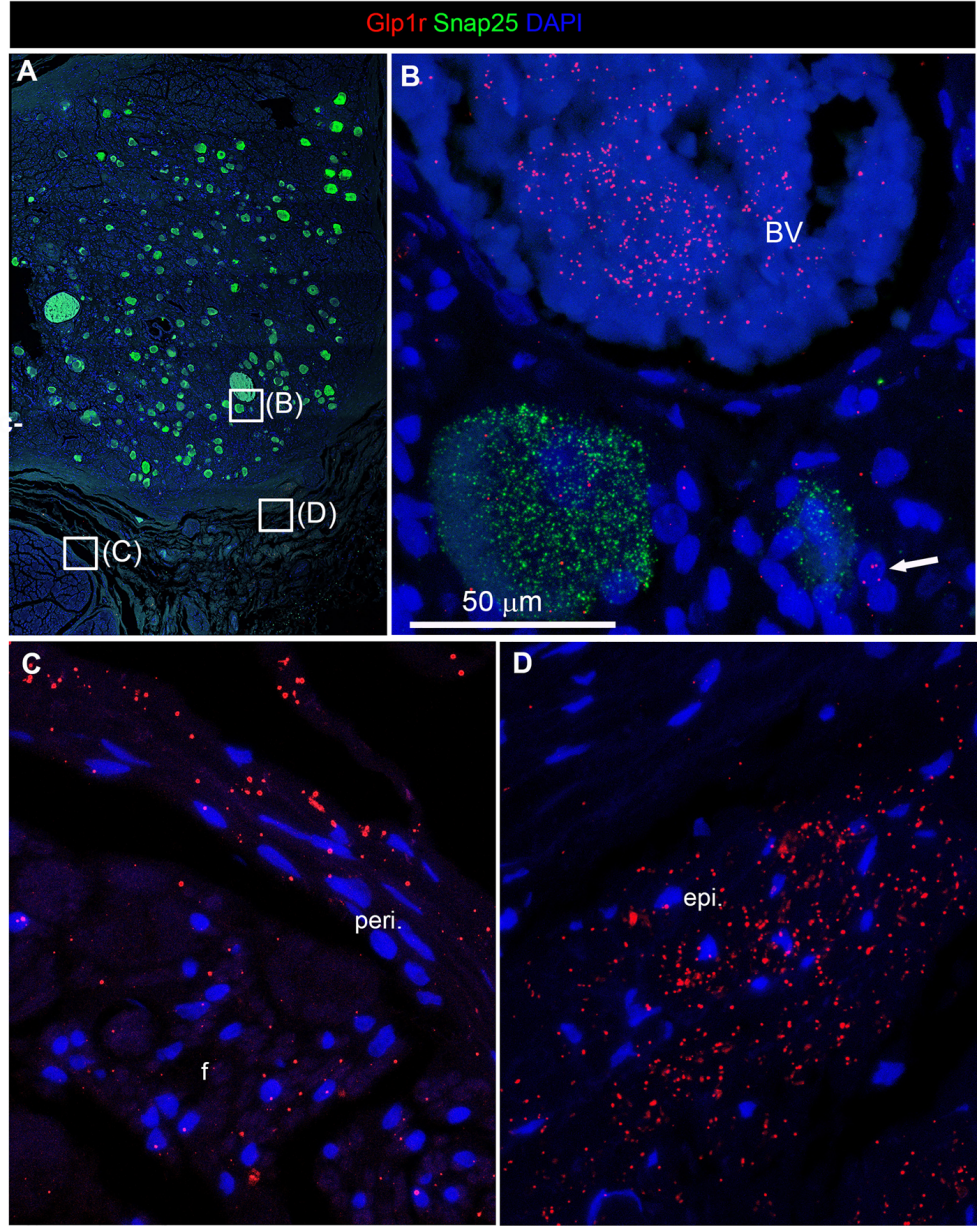
Representative confocal images showing *Glp1r* mRNA expression in nonneuronal cells of the human nodose ganglion. Samples were processed using multiplex RNAscope for *Snap25* (green), *Glp1r* (red), and DAPI (blue) and imaged with a Zeiss LSM980 confocal microscope. (A) Low‐magnification view (10x objective and tile imaging) of the nodose ganglion. Insets are shown at higher magnification (63x oil objective) in panels (B–D). Note that green autofluorescence originating from erythrocytes is observed in two large blood vessels. *Glp1r* signals were detected not only in *Snap25*‐positive neurons but also in small cells near the neurons (white arrow) that could be satellite cells, Schwann cells, or various immune cells (B). Notably, high *Glp1r* expression was consistently observed in the fibrous layers surrounding the nodose ganglion (peri‐ and epineurium), suggesting widespread nonneuronal expression (C, D). BV, blood vessels; f, fascicle; peri., perineurium; epi., epineurium.

In all samples, signals for *Glp1r* were consistently seen in nonneuronal cells near *Snap25*‐positive neurons (Figure 7A,B). Surprisingly, RNAscope signals were also seen in the lumen of large blood vessels with coagulated blood (Figure 7B). In contrast, RNAscope signals for Snap25 were not seen outside of neurons. Abundant expression was noted throughout the entire epineurium of the nodose ganglion and in the perineurium of vagal fascicles (Figure 7C,D). The signals were relatively uniform, suggesting that they corresponded to cells in connective tissues.

### 3.5 Comparison With Glp1r mRNA in the Mouse Nodose Ganglion

**FIGURE 8:**
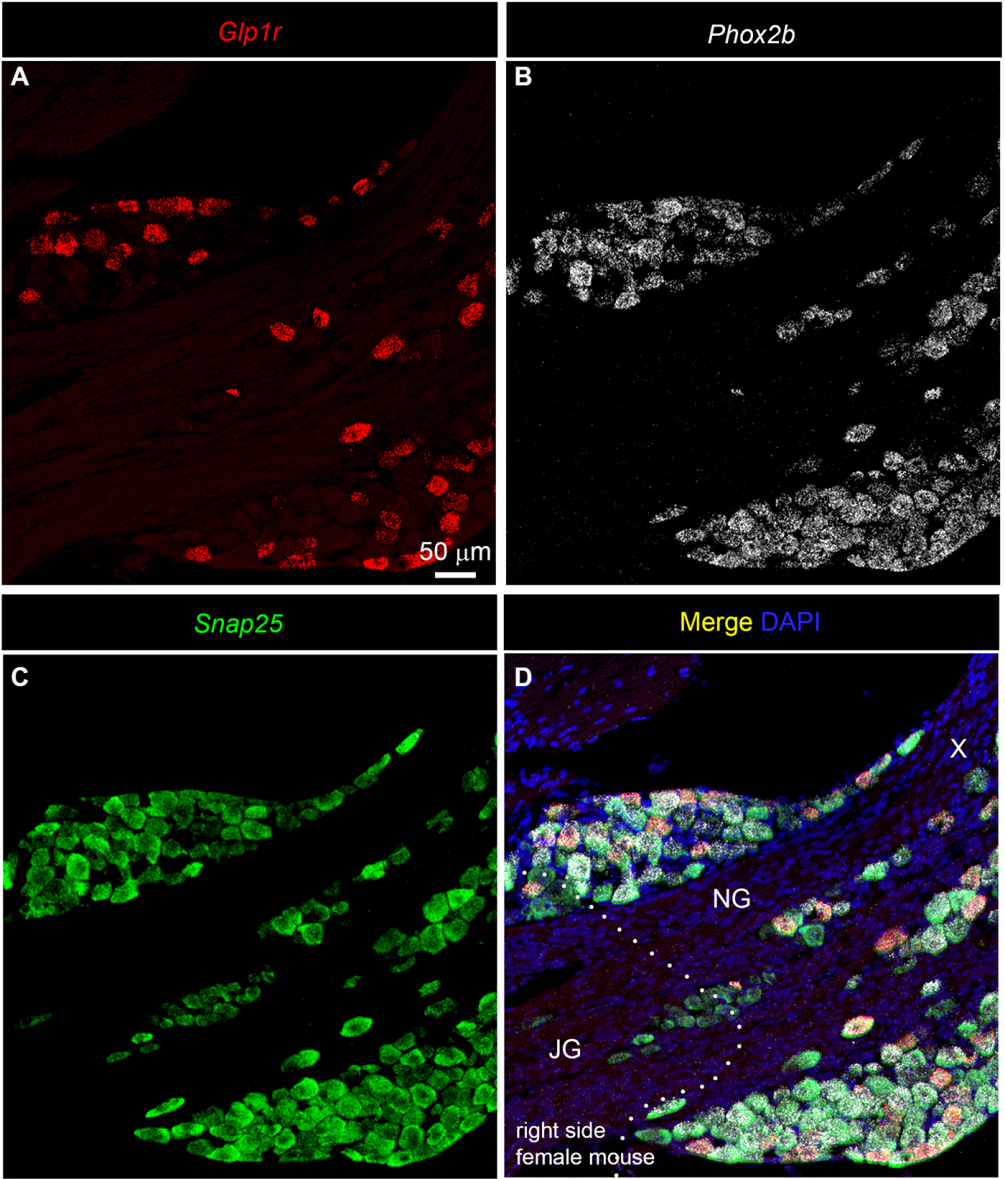
Digital images representative of the expression of *Glp1r* mRNA in the vagal ganglionic complex of a female C57Bl6/J mouse. Images were acquired using confocal microscopy (Zeiss LSM980) after processing samples with multiplex RNAscope in situ hybridization. (A) *Glp1r* signals (red) marked a large subset of vagal afferent neurons. (B) *Phox2b* signals (white) marked all vagal afferent neurons located in the nodose ganglion. (C) *Snap25* signals (green) marked all vagal afferent neurons located in both the nodose ganglion and the jugular ganglion. (D) Merged image combining transcripts, as well as counterstained with DAPI (blue). Please note how the left bottom portion of the ganglionic mass contains only *Snap25*‐positive neurons and likely corresponds to the jugular ganglia. In the rest of the ganglion, neurons were all positive for *Phox2b* and often co‐expressed *Glp1r*. No *Glp1r* signals were ever seen in the fibrous part of the ganglion. This ganglion corresponds to the anatomical right side. The dotted line separates the jugular ganglion from the nodose ganglion. Scale bar in (A) applies throughout. JG, jugular ganglion; NG, nodose ganglion; X, vagus nerve.

**FIGURE 9:**
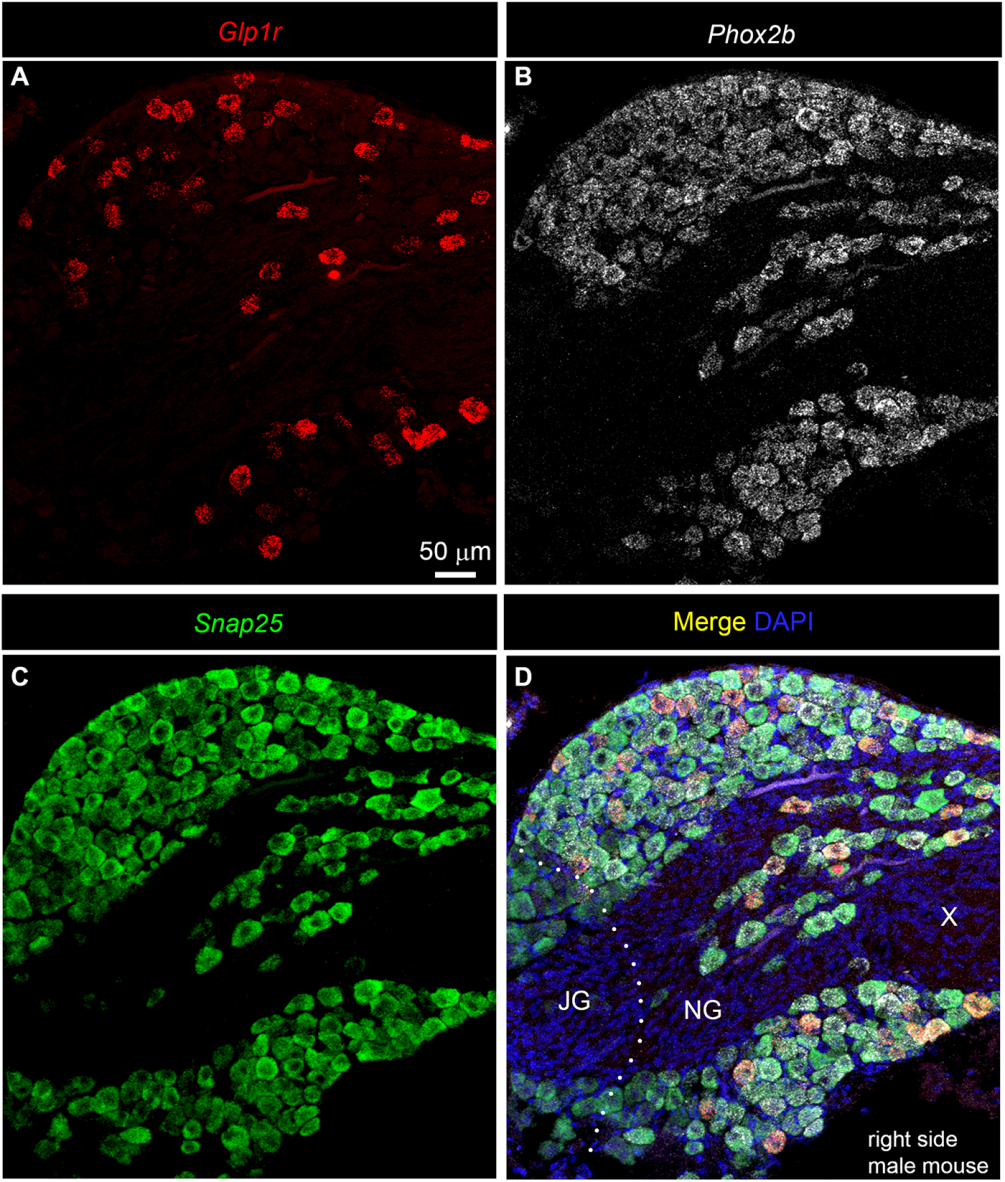
Digital images representative of the expression of *Glp1r* mRNA in the vagal ganglionic complex of a male C57Bl6/J mouse. Images were acquired using confocal microscopy (Zeiss LSM980) after processing samples with multiplex RNAscope in situ hybridization. (A) *Glp1r* signals (red) marked a large subset of vagal afferent neurons. (B) *Phox2b* signals (white) marked all vagal afferent neurons located in the nodose ganglion. (C) *Snap25* signals (green) marked all vagal afferent neurons located in both the nodose ganglion and jugular ganglion. (D) Merged image combining transcripts, as well as counterstained with DAPI (blue). Please note how the left bottom portion of the ganglionic mass contains only *Snap25*‐positive neurons and likely corresponds to the jugular ganglia. In the rest of the ganglion, neurons were all positive for *Phox2b* and often co‐expressed *Glp1r*. No *Glp1r* signals were ever seen in the fibrous part of the ganglion. This particular ganglion corresponds to the anatomical right side. The dotted line separates the jugular ganglion from the nodose ganglion. Overall, the distribution of Glp1r‐expressing cells in the male nodose ganglion resembled that of females. Quantitative data are provided in Figure 10. Scale bar in (A) applies throughout. JG, jugular ganglion; NG, nodose ganglion; X, vagus nerve.

After examining human samples, we next reexamined the distribution of *Glp1r* mRNA in the mouse nodose ganglion using a standard RNAscope multiplex procedure. Since the nodose ganglion is fused with the jugular ganglion in mice, we employed markers *Snap25* and *Phox2b* to differentiate neurons belonging to the jugular ganglion (*Snap25* ^+^/*Phox2b* ^−^) from those in the nodose ganglion (*Snap25* ^+^/*Phox2b* ^+^) (Kupari et al. 2019). In other words, the boundary corresponds to the transition zone between Phox2b^+^/Snap25^+^ cells and Snap25‐only cells. Consistent with previous reports (Lansbury et al. 2025; Leon‐Mercado et al. 2023; Williams et al. 2016), Glp1r expression followed an all‐or‐nothing distribution pattern, with a large subset of neurons expressing moderate to very high levels of *Glp1r*, while other neurons exhibited no expression (Figure 8). In both males and females, all *Glp1r*‐expressing neurons were also positive for *Snap25* and *Phox2b* and were restricted to the nodose ganglion (Figures 8 and 9). No *Glp1r* signals were observed in the epineurium, the fibrous component of the ganglion, or in the vagus nerve itself, confirming a strictly neuronal distribution of *Glp1r*.

**FIGURE 10:**
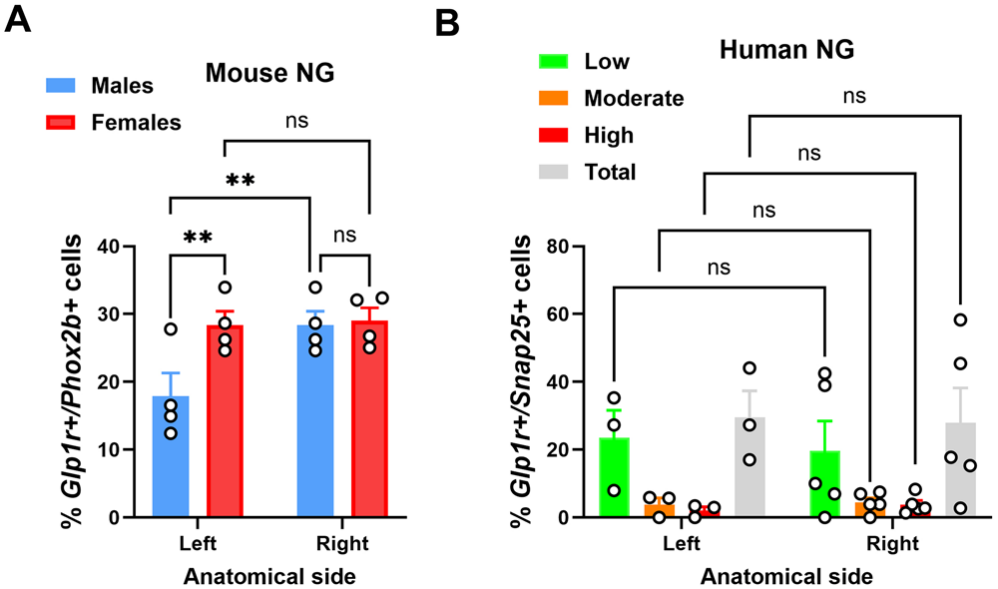
Comparison of the percentage of neurons expressing Glp1r in mice and humans. (A) Percentage of cells co‐expressing *Glp1r* and *Phox2b* in the mouse nodose ganglion (NG). Data are grouped by anatomical side and sex. Values are shown as mean ± SEM. A one‐way ANOVA followed by an uncorrected Fisher's LSD test was used for comparisons among all groups; **p < 0.01. Each dot represents one sample. (B) Percentage of cells co‐expressing *Glp1r* and *Snap25* in the human NG. In humans, *Glp1r*‐positive cells include low‐, moderate‐, and high‐expressing subpopulations (see Figure 6). Data are grouped by anatomical side and expression level. Human data were analyzed by two‐way ANOVA, followed by Sidak's multiple‐comparisons test; comparisons are made between anatomical sides. Each dot represents one sample. NS, not significant.

In females, our estimates showed that ∼24% and 29% of nodose neurons expressed *Glp1r* in the left and right ganglia, respectively (Figure 10A). In males, ∼18% and 28% of nodose neurons expressed *Glp1r* in the left and right ganglia, respectively (Figure 10A). These findings indicate a higher proportion of *Glp1r*‐expressing vagal afferents on the right side and suggest a degree of sexual dimorphism. However, while differences between anatomical sides in each sex reached statistical significance in males, no such differences were observed between sexes. In humans, regardless of *Glp1r* expression level, no difference between anatomical sides was observed (Figure 10B). Estimates indicated that approximately 29% of nodose neurons expressed *Glp1r* in both the left and right ganglia (Figure 10B).

## 4 Discussion

As noted in our introduction, nodose neurons expressing GLP1R are clearly involved in satiation. However, the specific role of GLP1R signaling in these neurons remains controversial due to discrepancies in the literature. In brief, studies deleting Glp1r from nodose neurons have reported divergent outcomes, with some finding an effect on feeding behavior and others not. Although our anatomical data cannot resolve the functional debate, they establish that vagal sensory neurons in human nodose ganglia are a potential target for the newly available GLP1R agonists. Whether GLP1R in these neurons contributes to metabolic effects and/or side effects remains to be verified. Interestingly, several qualitative and quantitative differences were observed between mouse and human samples. The cytoarchitecture of the human nodose ganglion differs markedly from that of rodents. In rodents, vagal sensory somata are tightly packed together, whereas in humans, they are more diffusely distributed. Additionally, the fibrous regions of the human ganglion are more voluminous, and the axonal bundles appear fragmented into larger fascicles. In addition, *Glp1r* mRNA was detected in cells of the peri and endoneurium of the vagus nerve in humans, a pattern not seen in mice. In our experiments, we consistently observed that *Snap25* signals were restricted to neurons, with no detectable expression in nonneuronal cells using either fluorescent or chromogenic approaches. Therefore, we do not believe that the RNAscope procedure itself is responsible for nonspecific background in nonneuronal cell types. Regarding GLP1R, transcriptomic datasets from the GTEX portal indicate expression across multiple human tissues, including high expression in the peripheral tibial nerves (https://gtexportal.org/home/gene/GLP1R). To us, this supports the possibility of genuine expression in nonneuronal cells within human nerves, although the specific cell types remain unknown. Based on our observations, GLP1R‐positive cells appear relatively widespread in the perineurial and epineurial layers and, to a lesser extent, within the nodose ganglion surrounding neurons. This pattern suggests a mixture of cell populations—potentially including perivascular cells, macrophages, glia, Schwann cells, and others. Because of the diversity of potential candidates, we have not yet pursued definitive identification. Future approaches such as spatial transcriptomics would be particularly well‐suited to address this question.

The human vagus nerve is known to exhibit asymmetry and sexual dimorphism (Biscola et al. 2024; Jayaprakash et al. 2023). The percentages of Glp1r‐positive nodose neurons reported by others (Lansbury et al. 2025; Welch et al. 2025) are not directly comparable to our counts because their data were not normalized to nodose‐specific markers such as Phox2b, but instead relied on more general neuronal markers encompassing both nodose and jugular populations. Nevertheless, both studies reported a higher number of *Glp1r*‐expressing cells on the right side, in agreement with our present findings. In contrast, no clear lateralization of *Glp1r* expression was observed in human samples; however, the limited sample size may have precluded definitive conclusions. Inherent interindividual variability in the human samples may also have contributed to potential counting bias. Additionally, we found that female mice exhibited the same number of *Glp1r*‐positive neurons compared to males. In human samples, sex‐based comparisons could not be made due to insufficient numbers of male donors.

Quantitative differences emerged between mice and humans. In human samples, *Glp1r* mRNA was expressed at lower levels in neurons than in mouse samples. For example, mouse neurons commonly showed moderate to high RNAscope signal that frequently filled the whole cell body, whereas human neurons displayed only a limited number of RNAscope puncta. Several explanations may account for these observations. First, the human nodose ganglion may be inherently more heterogeneous than its rodent counterpart and may dedicate fewer neurons to gastrointestinal innervation. Lower *Glp1r* levels in humans may also reflect species‐specific differences in receptor affinity, expression regulation, or endogenous GLP‐1 stability (Windelov et al. 2017).

Second, aging may have contributed to differences between mice and humans since we compared young animals to elderly humans. On the other hand, prior rodent studies suggest that the transcriptomic profile of vagal afferents is relatively stable across biological variables such as energy status, illness, age, or sex (McCoy and Kamitakahara 2023; Yuan et al. 2016). Accordingly, we do not expect age per se to have substantially impacted our results.

Third, we cannot fully exclude the possibility that certain medications and/or disease states may have influenced *Glp1r* expression in human sensory neurons (Hall et al. 2022). Unfortunately, we did not have access to the cause of death and detailed medical histories for our human donors, which represents a notable caveat. Nonetheless, we suspect that donor #82 had diabetes because of the presence of a permanent glucose sensor. Specimen #82 included a mixture of neurons: some exhibited normal morphology and *Snap25* expression, while others showed poor morphology and lacked *Snap25* expression, despite optimal preparation parameters. *Glp1r* was hardly detectable in samples from this donor. A likely explanation is the presence of neuropathy, causing cellular stress. If the patient had diabetes, this may represent the first demonstration of diabetes‐related vagal neuropathy in humans. In the DRG obtained from a human diabetic donor, histopathological structures called Nageotte nodules were previously identified (Shiers et al. 2025). However, we could not detect them in our nodose samples. Obtaining the nodose ganglion from young and healthy individuals would be useful, but such samples are difficult to come by.

### 4.1 Technical Considerations

Direct comparisons between mouse and human samples must be interpreted with caution. The two sample types could not be prepared using identical protocols, and methodological differences may also contribute, at least in part, to the discrepancies observed. Inherent technical limitations of human tissue studies—such as variability in PMI and fixation quality—could contribute to suboptimal detection of proteins and transcripts. While some degree of mRNA degradation is expected in human samples with a long PMI (Koppelkamm et al. 2011), the extent of this degradation in our samples remains uncertain. However, we were reliably able to detect *Snap25* at moderate to high levels across nearly all neurons, confirming the sensitivity of our assays. RNAscope has previously been used successfully with samples having a comparable or longer PMI than in the current study (Hurler et al. 2024), but we specifically selected samples with a PMI of approximately 10 h or less to optimize results. We found that the method is tolerant of PMIs up to 10 h, which is not surprising given that RNAscope probes are short and work in tandem, allowing for some degradation (Wang et al. 2012). Others have demonstrated that RNAscope generates robust signals even in human brain samples, which are mRNA‐degraded (Jolly et al. 2019). We are therefore confident in the sensitivity of our approach, even if it cannot be ruled out that low‐expressing *Glp1r* cells may have been lost. Notably, in our hands, samples with the longest PMI (>48 h) started to show signs of cell stress (if not death), including a smaller cell body and a large pericellular vacuolization. In a pilot study with mouse samples, we also found that drop fixation instead of perfusion minimally affects RNAscope results. Furthermore, a long PMI of up to ∼6 h did not modify *Glp1r* expression in the mouse nodose ganglion (data not shown).

It was also shown in the past that increased fixation times diminish the intensity of RNAscope signals (Colburn et al. 2024; Hurler et al. 2024). A 24‐h fixation period, which is the recommended duration by the RNAscope manufacturer, produces satisfying results with standard enzymatic pretreatment and extended boiling. However, a 48‐h fixation was also compatible with RNAscope, provided a longer enzymatic pretreatment of 45 min. It was reported that RNAscope signals can be detected in tissues stored for 15 years (Colburn et al. 2024). Our sections were collected within less than 1 year after resection and paraffin embedding, and hence, prolonged storage was not an issue.

In terms of specificity, we are confident that RNAscope provided unmatched specificity. This is because signals for *Snap25* and *Glp1r* are distributed according to expected patterns. For example, no signals for *Snap25* were seen outside of neurons. This is a major advantage over immunohistochemistry, considering that most commercial antibodies against receptors—including those against GLP1R—are unreliable (Gautron 2019; Michel et al. 2009; Wong et al. 2025).

One challenge is the presence of lipofuscin, a common artifact in older animals and human tissues, including sensory ganglia (Murakami et al. 2025; Sapio et al. 2025; Shiers et al. 2020). Since our human samples came from older donors, we took care to control this issue by illuminating the samples with a high‐power light, adjusting confocal parameters to minimize background autofluorescence capture. Overall, RNAscope signals remained easily distinguishable from lipofuscin.

The question of whether cells with only a few RNAscope dots should be considered positive for *Glp1r* remains a contentious issue. From a technical standpoint, any cell with detectable RNAscope dots can be classified as positive (Bono et al. 2022). However, it remains unclear whether these cells produce GLP1R at functionally meaningful levels. On the other hand, if cells with low expression are included, the proportion of positive cells rises to ∼39%, which is higher than that in mice. If only cells with moderate to high expression are included in our cell counts, the proportion of *Glp1r*‐positive cells is only ∼13%, which is lower than that in mice. This discrepancy raises the possibility that human nodose neurons inherently express fewer *Glp1r* transcripts or that these transcripts are more difficult to detect in thin paraffin‐processed, paraformaldehyde‐embedded (PPFE) sections compared to thicker, formalin‐fixed sections that were used in mice.

### 4.2 Conclusions

To our knowledge, *Glp1r* expression has not previously been investigated in the human nodose ganglion because of the difficulty in obtaining fresh samples, and therefore, no comprehensive studies of mRNA expression in the human nodose are available. We hope this study can start creating a molecular atlas of human vagal afferent subtypes, which could enhance our understanding of the vagus nerve as a therapeutic target. Our anatomical findings indicate that the human nodose ganglion is enriched in *Glp1r* mRNA, making it a potential target for the newly available GLP1R agonists. It would be interesting to examine whether the same *Glp1r*‐expressing subsets found in mice are present in humans. For example, it would be valuable to determine whether *Glp1r* in the human nodose ganglion colocalizes with the cholecystokinin receptor. Ultimately, spatial transcriptomics should be feasible and highly informative for defining these cell populations. In the future, it would also be interesting to investigate *Glp1r* expression in the nodose ganglion of cohorts with established obesity and diabetes diagnosis.

## Author Contributions

W.M. performed all the RNAscope studies. C.M. prepared the human tissue. C.G.J.C. collected human samples, contributed to study design, and edited the manuscript. L.G. designed the study, performed the microscopy, analyzed the data, and wrote the first draft of the manuscript.

## Funding

No funding was received for this research.

## Ethics Statement

Animal procedures were reviewed and approved by our Institutional Animal Care and Use Committee. Informed consent was obtained from human donors, and the study was reviewed and approved by the departmental research committee.

## Conflicts of Interest

The authors declare no conflicts of interest.

## Data Availability

All raw data and original images are available upon request.
